# Keeping an eye on pink eye: a global conjunctivitis outbreak expert survey

**DOI:** 10.1093/inthealth/ihab049

**Published:** 2021-08-19

**Authors:** Gurbani Kaur, Gerami D Seitzman, Thomas M Lietman, Stephen D McLeod, Travis C Porco, Thuy Doan, Michael S Deiner

**Affiliations:** University of California, San Francisco School of Medicine, San Francisco, CA 94143, USA; University of California, San Francisco Department of Ophthalmology, San Francisco, CA 94158, USA; University of California, San Francisco Department of Ophthalmology, San Francisco, CA 94158, USA; Francis I. Proctor Foundation for Research in Ophthalmology, San Francisco, CA 94158, USA; University of California, San Francisco Department of Ophthalmology, San Francisco, CA 94158, USA; Francis I. Proctor Foundation for Research in Ophthalmology, San Francisco, CA 94158, USA; University of California, San Francisco Department of Ophthalmology, San Francisco, CA 94158, USA; Francis I. Proctor Foundation for Research in Ophthalmology, San Francisco, CA 94158, USA; University of California, San Francisco Department of Ophthalmology, San Francisco, CA 94158, USA; Francis I. Proctor Foundation for Research in Ophthalmology, San Francisco, CA 94158, USA; University of California, San Francisco Department of Ophthalmology, San Francisco, CA 94158, USA; Francis I. Proctor Foundation for Research in Ophthalmology, San Francisco, CA 94158, USA; University of California, San Francisco Department of Ophthalmology, San Francisco, CA 94158, USA; Francis I. Proctor Foundation for Research in Ophthalmology, San Francisco, CA 94158, USA

**Keywords:** adenovirus, antimicrobial stewardship, conjunctivitis, EKC, epidemic, microbial surveillance

## Abstract

**Background:**

Recurrent conjunctivitis epidemics are prevalent worldwide. Aetiologies are often undetermined.

**Methods:**

We surveyed conjunctivitis researchers about perceived trends in prevalence, incidence and aetiologies of conjunctivitis epidemics.

**Results:**

Of the 155 participants, 7% endorsed globally variable and dynamic microbial aetiologies of conjunctivitis epidemics. Increased incidence of conjunctivitis epidemics over the last decade were reported by 21% of respondents. Peak seasons differed between the northern and southern hemispheres.

**Conclusions:**

There is regional equipoise regarding the increasing incidence and emerging underlying aetiologies of epidemic conjunctivitis. Further investigation of global surveillance and microbial characterization of conjunctivitis outbreaks could improve prevention and outcomes.

## Introduction

**Figure 1.**
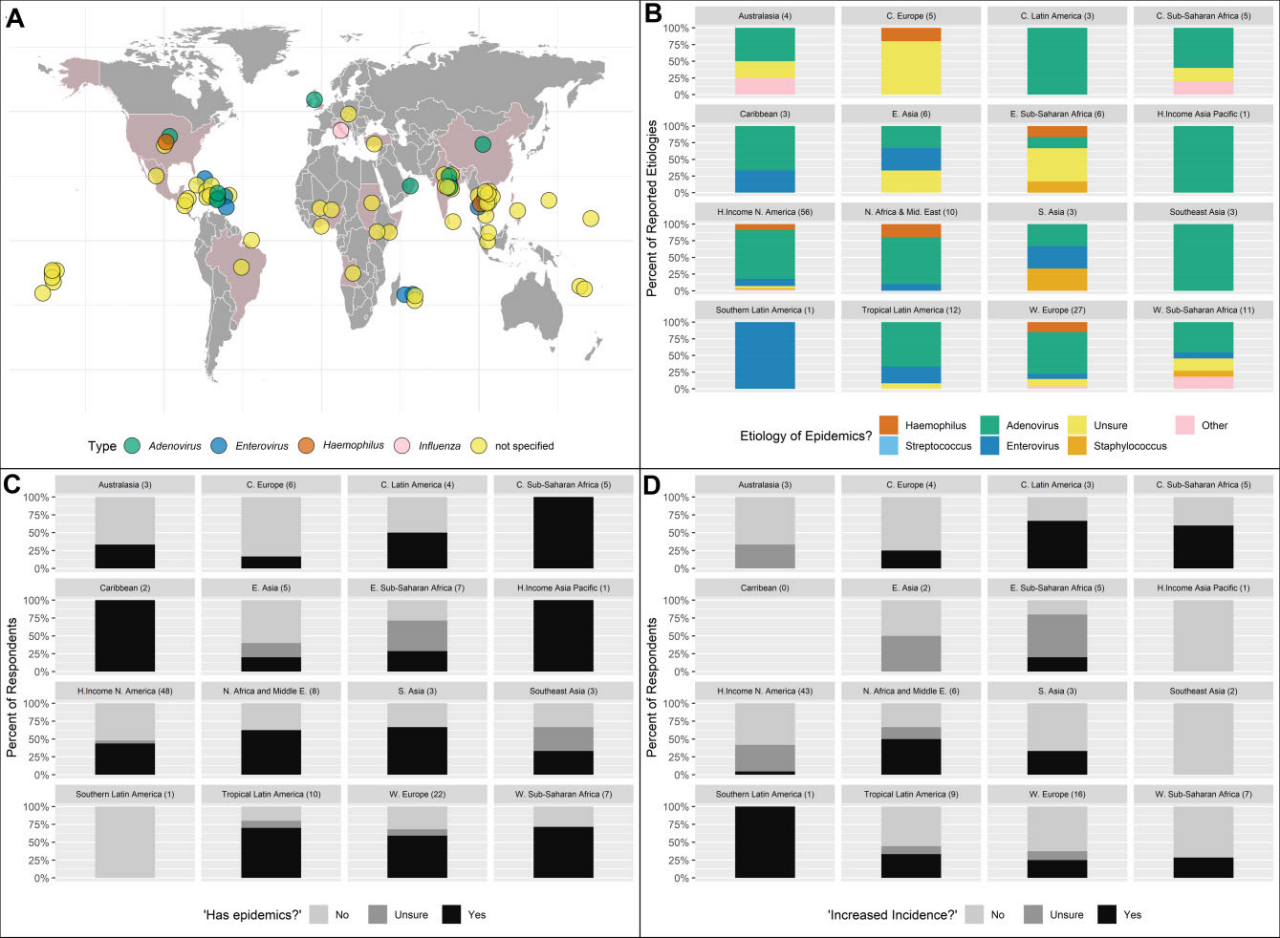
Reported trends in prevalence, aetiology and incidence of epidemic conjunctivitis. (A) Map depicting locations of conjunctivitis outbreaks from 2012 and 2017 as reported by PubMed, ProMED and other online news releases (section VIII. Supplement References from Supplementary Data^2^). Coloured circles indicate reported microbial aetiology or lack thereof (yellow). (B) Survey reported assumed microbial aetiology of international epidemics as a percentage of suspected pathogen by each GBD region. (C) Expert endorsement of prevalent conjunctivitis epidemics by GBD region as a percentage of expert responses by region. (D) Expert perception of increasing incidence of epidemics reported as a percentage of affirmation, ambivalence or denial by total responses in each GBD region. Sample sizes for each plot are indicated in parentheses in the label for each plot.

Conjunctivitis epidemics are common worldwide, afflicting people across age and socio-economic strata (Figure 1A). In the USA, conjunctivitis epidemics occur sporadically spatiotemporally. Non-US epidemics are often larger, with predictable patterns of seasonal outbreaks.^1^

The aetiologic pathogen for conjunctivitis is not typically identified. Frequently, providers assume adenoviral aetiology despite other well-documented viral, bacterial, fungal and allergic causes. Studying the pathogens and patterns of any infectious outbreak can provide insights into globally transmissible pathogens, both stable and emerging, and potentially improve prediction and treatment of future epidemics.

Herein we collected and compared worldwide conjunctivitis experts’ opinions on current aetiologies and characteristics of epidemics.

## Methods

Prior to our survey, for background and comparison to known prior outbreak patterns, we mapped (Figure 1A) locations and aetiologies of reported conjunctivitis outbreaks from 2012 to 2017 as reported by PubMed, ProMED and other online news releases^2^ (R packages ggplot, ggmap; R Foundation for Statistical Computing, Vienna, Austria).

We then performed a cross-sectional survey of conjunctivitis researchers who had published in a PubMed indexed journal since 2000 and of conjunctivitis experts associated with the Francis I. Proctor listserv. We used the R package easyPubMed to extract author e-mails, using a query requiring ‘conjunctivitis’ or ‘epidemic keratoconjunctivitis’ in any field and excluding references to animals. A total of 1950 experts received an e-mailed invitation and reminder to participate in the survey during the 1-week study period in September 2018. Using Qualtrics survey software (Qualtrics, Seattle, WA, USA), respondents provided perceived trends over the last 10 y in the prevalence, incidence and aetiology of conjunctivitis epidemics in their primary geographic location of expertise. Testing for homogeneity was conducted using clustered logistic regression, adjusting for the occurrence of the null hypothesis on the boundary of the parameter space.^3^

## Results

A total of 155 survey responses were collected for analysis, representing a response rate of 7.9%. Locations of respondents are shown in Figure 1B–D, grouped by Global Burden of Disease (GBD) region.^4^ Of the 21 GBD regions, responses from 16 were represented in this report (Figure 1B–D). Regional conjunctivitis epidemics were reported by 51% of respondents. The probability of claiming epidemics did not differ between GBD regions (p=0.34, clustered logistic regression). Of respondents reporting epidemics, 75% recorded a presumed adenoviral aetiology. The probability of claiming adenoviral aetiology was not homogeneous between GBD regions (p<0.001, clustered logistic regression; Figure 1B). ‘Unknown’ as an aetiology of conjunctivitis epidemics was reported in 9 of 11 GBD regions (Figure 1B). The presence of conjunctivitis epidemics was reported in 15 of 16 of the represented GBD regions (Figure 1C), with consistent regional endorsement by experts in high-prevalence regions: central sub-Saharan Africa, the Caribbean and high-income Asia Pacific. An increasing incidence of infectious conjunctivitis over the past decade was endorsed by 21% of participants worldwide (3% of US participants; see Figure 1D) with most experts citing increasing incidences in Latin America, central sub-Saharan Africa and southern Latin America. Overall, 7% of participants globally (3% of US participants) believed the aetiology of these epidemics is variable and changing. Using permutation testing, the peak conjunctivitis season differed between the northern and southern hemispheres (p=0.004). Southern hemisphere countries reported a greater incidence of outbreaks during months of the typical US low season of late summer to early fall.

## Discussion

This study was conducted to determine if there is a global consensus among those who study and treat conjunctivitis epidemics. Experts confirmed that global conjunctivitis epidemics are prevalent and suspected aetiologies are largely unknown or variable. When specific aetiologies were indicated, experts did not exclusively attribute conjunctivitis epidemics to adenoviral origin. This is consistent with prior reports indicating a lack of consensus on outbreak patterns and aetiologies.^2^ Uncertainty regarding aetiology is expected to lead to misdiagnosis and improper treatment that contribute to billions expended globally due to costs of medication and of missed work and school.^5^ Presumptive treatment of viral conjunctivitis with antibiotics does not benefit patients. This practice contradicts antibiotic stewardship and endangers patients and populations by fuelling antibiotic resistance.^6^ Guidance to curb wasteful spending and antibiotic resistance while improving outcomes cannot be implemented absent elucidation of regional epidemic aetiologies. Additionally, incorrect assumptions that infectious conjunctivitis has self-limited viral aetiologies could result in misdiagnoses, sometimes with missed systemic disease implications. A lack of systematic conjunctivitis surveillance and diagnostic microbial confirmation in routine clinical practice and in public health efforts contributes to this paucity of information.

Limitations of any survey study include recall bias and subjectivity. Another limitation of this report is a low response rate, which could increase the likelihood of bias in some direction. Potential contributors to our response rate included international variability to e-mail access, language barriers, participation apathy and lack of representation from five GBD regions.

The reported equipoise regarding the increase, seasonality and underlying aetiologies of conjunctivitis by region warrants further investigation to determine if global surveillance and microbial characterization of conjunctivitis outbreaks can improve prevention and outcomes. It's time to keep an eye on pink eye.

## Data Availability

De-identified data are available in the article and in its online supplementary material.
